# EMERGENCE OF ADDICTION SUBSTITUTION: SUBSTANCE USE RESULTING FROM ALCOHOL USE DISORDER RECOVERY IN A US SAMPLE

**DOI:** 10.1093/geroni/igad104.2515

**Published:** 2023-12-21

**Authors:** Yixuan Sun, Thomas Kwan, Douglas Bowlby, Matthew Lee

**Affiliations:** Rutgers University New Brunswick, Piscataway, New Jersey, United States; Rutgers University New Brunswick, Piscataway, New Jersey, United States; Rutgers University, New Brunswick, New Jersey, United States; Rutgers University New Brunswick, Piscataway, New Jersey, United States

## Abstract

Addiction substitution refers to the increased use of other addictive substances after recovery from a prior addiction. Previous research shows that addiction substitution may lead to a poorer recovery prognosis, and an increased chance of relapse with the primary substance. If health concerns affect the likelihood of substance switching, then this concerns aging segments of the U.S. population. The aim of this study was (1) to examine the relationship between recovery from alcohol use disorder (AUD) and the subsequent use of other substances and (2) to identify covariates that affect the relationship between recovery and addiction substitution. This study used two-waves of longitudinal data from NESARC (National Epidemiologic Survey on Alcohol and Related Conditions), a U.S. representative sample aged 18-90+. We used 2*2 Chi-square analyses to examine the likelihood of the emergence of Wave 2 substance use (e.g., opioids) among those who had recovered from AUD between Waves 1 and 2. We ran a subsequent analysis to determine if various health outcomes predicted substance switching. The results showed that there was a significant decrease in the likelihood of becoming an opioid user by wave 2 among those who recovered from AUD in wave 1. Analysis of potential confounds identified that those who recovered from AUD but reported poor health were more likely than other recoveries to increase opioid use. Results suggest that recovery from AUD reduces the use of other substances. However, health issues increase the likelihood of substance switching occurring among those recovering from AUD.

